# Anthropometric Study of the Human Ear Among Patients Visiting People’s Dental College and Hospital: An Observational Study

**DOI:** 10.31729/jnma.v63i292.9249

**Published:** 2025-12-31

**Authors:** Mangesh Bajracharya, Shyam Kaji Maharjan, Dristi Shrestha, Chetana Pathak

**Affiliations:** 1 Department of Anatomy, People’s Dental College and Hospital, Nayabazar, Kathmandu, Nepal; 2 Department of Prosthodontics, People’s Dental College and Hospital, Nayabazar, Kathmandu, Nepal; 3 Department of ENT, People’s Dental College and Hospital, Nayabazar, Kathmandu, Nepal

**Keywords:** *anthropometry*, *auricular dimensions*, *sexual dimorphism*

## Abstract

**Introduction:**

Anthropometric measurements of the external ear provide valuable normative data for applications in clinical practice, reconstructive surgery, forensics, and ergonomics. As ear morphology varies by population, sex, and age, population-specific reference data are essential. This study aimed to determine the normal dimensions of the outer ear among adult patients.

**Methods:**

A cross-sectional study was conducted from 8th to 28th September 2025 among 157 adult patients (82 males and 75 females) visiting the hospital with normal external ears. Standardized auricular measurements, including auricular height, auricular width, lobular height, and lobular width, were obtained bilaterally using a digital vernier caliper following established anthropometric landmarks. Data were analyzed using descriptive statistics to calculate the mean and standard deviation.

**Results:**

The mean right and left auricular heights were 63.38 ± 6.19 mm and 63.13 ± 6.49 mm, respectively, while auricular widths were 33.81 ± 6.26 mm (right) and 33.82 ± 6.23 mm (left). Minimal bilateral asymmetry was observed. Males exhibited greater auricular height and width, whereas females showed marginally larger lobular dimensions. Lobular height demonstrated greater variability than width across both sexes.

**Conclusion:**

The study revealed strong bilateral symmetry in external ear measurements, with clear sexual dimorphism—males having larger auricular dimensions and females slightly larger lobules. This finding provide baseline data for Nepalese adults and hold relevance in forensic identification, prosthetic design, and reconstructive surgery. Further large-scale, multiethnic studies are recommended to validate and expand upon these findings.

## INTRODUCTION

Anthropometry is the scientific measurement of human body measurements that provides important normative information for clinical practice, forensic identification, ergonomics and biomedical device design.^1^

The external ear is an anthropometrically informative structure because its measurable dimensions have population-specific patterns and useful sex- and age-based differences which inform reconstructive surgery, prosthetic design and biometric and forensic use.^2^ Many studies report population-specific anthropometric values in different countries including confirming persistent sexual dimorphism, some degree of bilateral asymmetry, and age-related changes.^3^

Therefore, an anthropometric study of the external ear is needed in order to acquire baseline data for a specified population which can be utilized in clinical, forensic, and anthropological uses. The present study attempted to determine the normal dimensions of the outer ear in the patients since limited research^4-6^ especially among the students has thus far been conducted in our country.

## METHODS

This cross-sectional study was conducted in the patients visiting People’s Dental College and Hospital, Nayabazar, Kathmandu, from 8^th^ September to 28^th^ September 2025. The outpatient department at the study site receives 30 to 40 patients daily. Data collection was started after getting the ethical clearance from the Institutional Review Committee of People’s Dental College and Hospital (Ref. No: CH no 3,2082/2083). The total population considered for the study was all patients visiting the outpatient department during the study period. Of these, 15-20 patients were selected each day according to the inclusion criteria, out of which convenient sampling was used to obtain the sample population. As an inclusion criterion, all patients 18 years and older attending outpatient departments with both normal external ears were eligible for the study. Those patient with congenital auricular abnormalities, tumors, previous surgical history, and ear piercing done were excluded from the study.

The sample size was calculated by using the formula: Z^2^ ^2^\d^2^, where “Z” value is 1.96 at 95% confidence interval. “” value is standard deviation=3.91^5^; “d” value represents the margin of error is calculated as 1% of 61.29^5^= 0.61. The total number of patients for the study was calculated as 157. Subsequently, the written informed consent of all participants was taken during the conduct of the study. Bilateral sizes of auricles were measured in millimeters by using a digital vernier caliper. Standardized measurements of the ear auricles were taken according to the landmarked points defined by De Carlo et al^7^ and the methodology was adopted from McKinney et al^8^ and Brucker et al.^9^ With participant kept in the comfortable position and the head was positioned in the Frankfort horizontal plane the parameters measured were ear height, ear width, lobular height, and lobular width for each participant’s right and left ears. The ear height was measured as the distance from the most inferior projection of the ear lobule to the most superior projection of the helix. The ear width was measured as the distance between the most anterior and posterior points of the ear. The lobule height was taken as the distance from the most inferior end of the lobule to the base of the tragal notch. The lobule width was measured as the horizontal width of the lobule.

All the measurements were recorded in the proforma and transformed to excel sheet in the password-protected laptop. Statistical analysis was done in IBM SPSS Version 16. Descriptive statistics such as mean, standard deviation (SD) were determined.

## RESULTS

A total of 157 patients were included in the study out of which 82 (52.20%) were male and 75 (47.80%) were female. The median age of the participants was 40 years (IQR: 19-62 years). Descriptive analysis was performed to calculate the mean and standard deviation of parameters of the total population (n=157) (Table 1) The mean right auricular height was calculated as 63.38 ± 6.19 mm and left auricular height as 63.13 ± 6.49 mm, while auricular width was 33.81 ± 6.26 mm for right and 33.82 ± 6.23 mm for the left. Lobular size also demonstrates minimal asymmetry, with the lobular height as 23.06 ± 8.21 mm and lobular width as 22.29 ± 4.42 mm of the right side being minimally larger than the left lobular height: 22.50 ± 8.01 mm and left lobular width: 21.65 ± 3.74 mm.

Similarly, the mean and standard deviation of the parameters were calculated for male (n=82) and female (n=75) (Table 2).

**Table 1:** Descriptive analysis of parameters in total population (n=157).

Parameters	Mean±SD (mm)
Right Auricular Height	63.38±6.19
Right Auricular Width	33.81±6.26
Right Lobular Height	23.06±8.21
Right Lobular Width	22.29±4.42
Left Auricular Height	63.13±6.49
Left Auricular Width	33.82±6.23
Left Lobular Height	22.50±8.01
Left Lobular Width	21.65±3.74

**Table 2:** Mean and S.D of male and female patients (n=157).

	Males (n=82)	Females (n=75)
Parameters	Mean±S.D	Mean±S.D
Right Auricular Height	64.17±5.64mm	62.52±6.66mm
Right Auricular Width	34.75±6.18mm	32.78±6.22mm
Right Lobular Height	22.68±7.19mm	23.47±9.23mm
Right Lobular Width	21.88±4.79mm	22.74±3.96mm
Left Auricular Height	63.87±5.84mm	62.32±7.08mm
Left Auricular Weight	34.80±6.13mm	32.76±6.21mm
Left Lobular Height	22.16±7.03mm	22.88±9.00mm
Left Lobular Width	21.18±3.90mm	22.17±3.5mm

Auricular sizes were calculated. Right auricular height (males: 64.17 ± 5.64 mm; females: 62.52 ± 6.66 mm) and left auricular height (males: 63.87 ± 5.84 mm; females: 62.32 ± 7.08 mm) with higher values in males. Also, there is a similar trend in auricular width (males: right 34.75 ± 6.18 mm, left 34.80 ± 6.13 mm; females: right 32.78 ± 6.22 mm, left 32.76 ± 6.21 mm. Conversely, females have slightly larger lobular sizes: right lobular height (23.47 ± 9.23 mm) in compare to males (22.68 ± 7.19 mm) and lobular width with 22.74 ± 3.96 mm in female in compare to 21.88 ± 4.79 mm in male. Similar trend was observed on the left side too with lobular height as 22.88 ± 9.00 mm in female compare to 22.16 ± 7.03 mm in male; lobular width as 22.17 ± 3.50 mm in female compare to 21.18 ± 3.90 mm in male.

## DISCUSSION

Deformity of the external ear may be a consequence of genetic disease or environmental trauma due to injury, infection, radiation, and other factors. Such auricular deformities can be aesthetically displeasing and may lead to social withdrawal or reduced self-esteem, ultimately affecting the psychological and social well-being of the individual. Otoplastic surgery can correct congenital auricular defects or reconstruct deformities caused by trauma, emphasizing the importance of precise anatomical data for achieving symmetry and natural appearance.^10^ Malformations of the external ear can also result from genetic disorders or environmental damage caused by factors such as trauma, infection, and radiation.^11^ Thus, the morphometric values of various auricular points in various age and gender groups have become crucial to know, for precise plastic reconstruction.^12^ A study among Nepalese medical students aged 18-21 reported mean external ear dimensions, revealing that males had significantly larger total ear length, ear width, lobular length, and lobular width than females, this pattern corresponds with the findings of the present study, which also demonstrated larger auricular height and width in males relative to females. Interestingly, while the earlier research observed that all measured parameters were greater on the right ear than on the left suggesting a mild right-side dominance, our study revealed only minimal right-left differences, indicating a more balanced bilateral symmetry among the broader adult population sampled. This contrast may reflect age-related stabilization of auricular growth, differences in measurement methodology, or population heterogeneity beyond the student demographic.^4^ Further comparative insight is offered by the anthropometric study conducted by Gupta and Ambekar, which sought to establish normative auricular measurements among 200 medical students, comprising 134 Nepalese and 66 Indian participants aged 18 to 24 years. Their results revealed clear inter-ethnic variation: Indian male students displayed greater auricular height and lobular height than their Nepalese counterparts, although auricular width did not differ significantly between the two groups. Notably, lobular width demonstrated a side-specific difference, being larger on the left in Indian males, while showing parity on the right side. Among females, all measured auricular parameters were consistently larger in the Indian group compared to the Nepalese participants, reinforcing the influence of both sex and ethnicity on external ear morphology. When analyzed for sexual dimorphism within each ethnic group, males consistently exhibited larger auricular dimensions than females, a trend that underscores the biological influence of sex on ear growth and morphology. The reported mean auricular height among Indian males was approximately 63.23 mm bilaterally, compared with 61.35 mm in Nepalese males, while female Indian participants exhibited mean auricular heights of about 60.23 mm (right) and 60.19 mm (left), both exceeding those of Nepalese females at 57.23 mm and 57.56 mm, respectively. These findings suggest that population-specific factors such as genetic makeup, nutritional status, climatic adaptation, and craniofacial morphology may contribute to subtle variations in auricular size and shape. Taken together, these comparative studies highlight the significance of establishing localized anthropometric reference standards. Differences in ear morphology between Nepalese and Indian populations and even between subgroups within Nepal, underscore the role of ethnic, genetic, and environmental determinants in shaping external ear dimensions. The consistency of male predominance in auricular height and width across studies reinforces the biological basis of sexual dimorphism, while variations in bilateral asymmetry suggest that population-specific developmental patterns and sampling characteristics may influence auricular morphology.^5^ The current study’s findings of bilateral symmetry in auricular dimensions (minimal right-left differences less than 0.3 mm overall; less than 0.5 mm by sex) align with the anthropometric investigation conducted on Korean adults showing disparities of 0.6-1.3 mm.^13^ Studies in North Indian and Nepalese medical students also found comparable symmetry, with minor right-side dominance in Auricular height and width, consistent with our data and underscoring the reliability of measurements for reconstructive purposes.^14, 15^ In diverse populations, males exhibit larger auricular height (approximately 1.5 to 2 mm greater) and width, whereas females show marginally larger lobular dimensions, less than 1 mm. Study conducted on Maharashtrian population in India, have confirmed that males have larger auricular sizes, although the results for lobular sizes are variable, with some females displaying wider lobes.^16^ While Saudi Arabian and Indonesian research highlights overall male dominance in ear metrics for forensic sex estimation.^17, 18^ The mean auricular height in the present study appear larger than study performed by previous authors in Nepalese population, these discrepancies could be a result of factors such as race, genetic variables, individual constitution, environment, age, and human error.^4-6, 15^ The findings of this research are directed towards significant uses in a range of fields of application such as forensic identification, ergonomics, and reconstructive surgery. Within forensic science, the calculation of normative auricular dimensions can be a very valuable means of personal identification, particularly where other biometric markers are not present. Because auricular features are fairly stable in adulthood and exhibit population-dependent variations, they can contribute to sex identification and population determination if utilized in conjunction with other morphological or genetic features. In product design and ergonomics, having an awareness of average ear sizes makes it easier to design hearing aids, earphones, helmets, and other wearables that provide precise fitting, comfort, and functionality for the target population. From a reconstructive and aesthetic surgical perspective, such normative values provide key points of reference for reconstruction or reshaping auricular anatomy following congenital anomalies or trauma, improving surgical results and facial harmony. However, the relatively small sample size and absence of precise demographic details like stratification by age, ethnic subgroups, and regional distribution rule out generalization of these findings. These factors can influence auricular morphology and might not have been fully held constant in the present study. In comparison with larger-scale analyses, our measurements validate worldwide preservation of auricular symmetry but observe greater variability in lobular size, possibly reflective of genetic heterogeneity, adaptation to the environment, or age-related changes in morphology, particular to this sample. Thus, coming studies must include larger, demographically representative populations and employ advanced morphometric and statistical analyses to replicate these patterns, explore their functional and developmental significance, and confirm their validity for application in clinical, ergonomic, and forensic practice.

## CONCLUSION

The present study found strong bilateral symmetry in ear dimensions, with right-left sides differences. Males exhibited slightly larger auricular height and width than females. While females showed marginally larger lobular dimensions. Lobular height displayed greater variability than width. The results have implications for forensics, ergonomics, and otoplasty. The limited sample size and lack of detailed demographic data highlight the need for larger, more diverse studies to enhance applicability.

## Data Availability

The data are available from the corresponding author upon reasonable request.
